# Polyunsaturated fatty acids in various macroalgal species from north Atlantic and tropical seas

**DOI:** 10.1186/1476-511X-10-104

**Published:** 2011-06-22

**Authors:** Vincent JT van Ginneken, Johannes PFG Helsper, Willem de Visser, Herman van Keulen, Willem A Brandenburg

**Affiliations:** 1 Plant Research International, Business Unit Agrosystems Research, P.O. Box 616, 6700 AP Wageningen, The Netherlands; 2 Plant Research International, Business Unit Bioscience, P.O. Box 619, 6700 AP Wageningen, The Netherlands

## Abstract

**Background:**

In this study the efficacy of using marine macroalgae as a source for polyunsaturated fatty acids, which are associated with the prevention of inflammation, cardiovascular diseases and mental disorders, was investigated.

**Methods:**

The fatty acid (FA) composition in lipids from seven sea weed species from the North Sea (*Ulva lactuca, Chondrus crispus, Laminaria hyperborea, Fucus serratus, Undaria pinnatifida, Palmaria palmata, Ascophyllum nodosum*) and two from tropical seas (*Caulerpa taxifolia, Sargassum natans*) was determined using GCMS. Four independent replicates were taken from each seaweed species.

**Results:**

Omega-3 (n-3) and omega-6 (n-6) polyunsaturated fatty acids (PUFAs), were in the concentration range of 2-14 mg/g dry matter (DM), while total lipid content ranged from 7-45 mg/g DM. The n-9 FAs of the selected seaweeds accounted for 3%-56% of total FAs, *n*-6 FAs for 3%-32% and *n*-3 FAs for 8%-63%. Red and brown seaweeds contain arachidonic (C20:4, n-6) and/or eicosapentaenoic acids (EPA, C20:5, n-3), the latter being an important "fish" FA, as major PUFAs while in green seaweeds these values are low and mainly C16 FAs were found. A unique observation is the presence of another typical "fish" fatty acid, docosahexaenoic acid (DHA, C22:6, n-3) at ≈ 1 mg/g DM in *S. natans*. The n-6: n-3 ratio is in the range of 0.05-2.75 and in most cases below 1.0. Environmental effects on lipid-bound FA composition in seaweed species are discussed.

**Conclusion:**

Marine macroalgae form a good, durable and virtually inexhaustible source for polyunsaturated fatty acids with an (n-6) FA: (n-3) FA ratio of about 1.0. This ratio is recommended by the World Health Organization to be less than 10 in order to prevent inflammatory, cardiovascular and nervous system disorders. Some marine macroalgal species, like *P. palmata*, contain high proportions of the "fish fatty acid" eicosapentaenoic acid (EPA, C20:5, n-3), while in *S. natans *also docosahexaenoic acid (DHA, C22:6, n-3) was detected.

## Background

Polyunsaturated fatty acids (PUFAs) are essential nutrients which cannot, or only to a limited extent, be synthesised by mammals. Therefore, they must be ingested via dietary sources [1,2]. The two main PUFA classes are omega-3 (n-3) and omega-6 (n-6). The n-3 PUFAs are provided by fish and plant sources, whereas the n-6 PUFAs are ingested mainly via vegetable oil [2,3].

Degenerative diseases related to inappropriate fatty acid consumption form a major, potential death cause for two thirds of the population living in affluent, industrialised nations [4]. Sixty eight percent of the people die from three conditions which involve fatty acid (FA) degeneration: cardiovascular disease (43.8%), cancer (22.4%), and diabetes (1.8%) [5,6].

**Figure 1:**
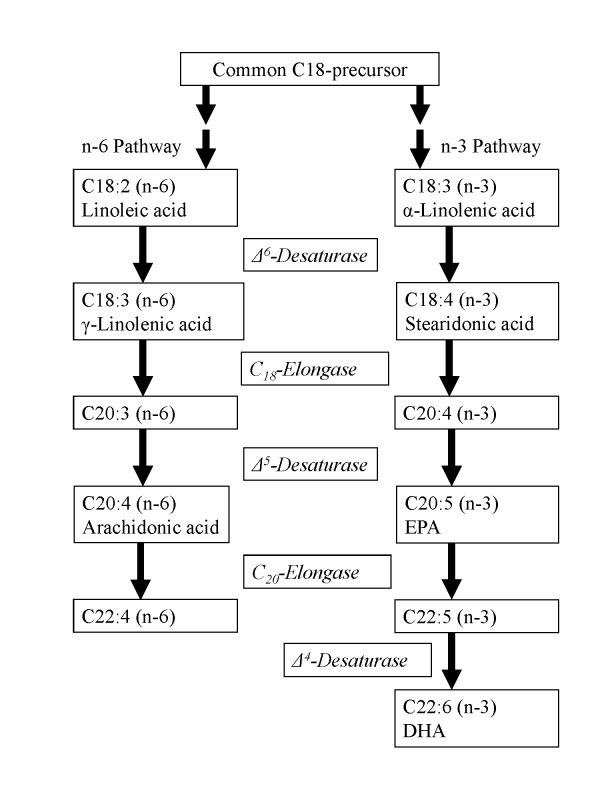
**Biosynthetic pathway for the dietary most important long-chain n-6 and n-3 polyunsaturated fatty acids**.

Two PUFAs which cannot be synthesized by humans and other vertebrates are linoleic acid (C18:2, n-6) and α-linolenic acid (C18:3, n-3). The PUFAs include two metabolic series of compounds: the n-6 and the n-3 FAs. Linoleic acid belongs to the n-6 series while linolenic acid refers to both α-linolenic (C18:3, n-3) and γ-linolenic acid (C18:3, n-6). Within the body both can be converted to other PUFAs such as arachidonic acid (C20:4, n-6), eicosapentaenoic acid (EPA, C20:5, n-3) and docosahexaenoic acid (DHA, C22:6, n-3). There are two pathways for the conversion of C18 PUFAs to long chain PUFAs: Linoleic acid is converted to arachidonic acid in the n-6 series and α-linolenic acid is converted to EPA and DHA in the n-3 series (Figure 1, reviewed [3,7]).

It is important to maintain an appropriate balance of n-3 and n-6 in the diet as these FAs work together to promote health: n-3 FAs have been recognised to exhibit anti-inflammatory and antioxidant activity, which may contribute to their beneficial cardiac effects [3,8,9], but also to the prevention of breast cancer [10]. In contrast, most n-6 FAs (precursors of arachidonic acid and prostaglandin E_2_) tend to promote inflammation and tumour growth [11,12]. Recently, it became clear that besides prevention of cardiovascular diseases [9,13,14] some n-3 PUFAs, especially EPA and DHA, are major components of brain cells and crucial for proper development and functioning of the brain and the nervous system [15,16]. Also with a world-wide increase in lifetime expectancy [17] it is an important observation that dietary n-3 fatty acid supplementation to the elderly results in an increased muscle protein synthesis, in this way preventing sarcopenic obesity [18].

Until now the major source of n-3 and n-6 long-chain PUFAs, such as arachidonic acid, EPA and DHA, is fish oil [19]. However, it is noteworthy that the original source of these long-chain PUFAs is not the fish itself, but marine algae and phytoplankton which form their major dietary source [20].

A recent study [21,22] predicts a rapid worldwide depletion of fish populations. Already 29% of edible fish and seafood species has declined by 90%, which indicates a collapse of fisheries and salt-water fish extinction by the year 2048 [22]. Therefore, other sources for n-3 and n-6 FAs have to be found. Seaweeds are abundant and poorly exploited. Three major groups of seaweeds can be distinguished: Chlorophyta (1200 species), Phaeophyceae (2000 species) and Rhodophyta (6000 species) [23]. Although in general their lipid content is low we hypothesise that n-3 and n-6 PUFAs can be extracted from these macroalgae.

In addition, the n-6: n-3 ratio, which is currently recommended by the WHO [24] to be lower than 10 in the diet, can possibly be improved by addition of certain edible seaweeds because of their high n-3 content.

## Methods

### Seaweeds

Fronds of nine different species of benthic marine macrophytes (macroalgae) with different growth strategies and morphologies were collected from the upper and mid-littoral zone in September and October 2009. Four replicates were sampled for every seaweed species on each location. The seaweed species collected at the various locations were:

- *Ulva lactuca *(Chlorophyta): Katse Heule, Eastern Scheldt, the Netherlands. Approximate coordinates: 51°32'30 N and 3°52' E

- *Laminaria hyperborea *(Phaeophyceae), *Chondrus crispus *(Rhodophyta), *Fucus serratus *(Phaeophyceae): Concarneaux, France. Approximate coordinates: 47°52' N and 3°55' W

- *Palmaria palmata *(Rhodophyta), *Undaria pinnatifida *(Phaeophyceae): Kilcar, West Donegal, Ireland. Approximate coordinates: 54°37' N and 8°37'W

- *Ascophyllum nodosum *(Phaeophyceae): Island Solund, Norway. Approximate coordinates: 61°03' N and 4°52' E

- *Sargassum natans *(Phaeophyceae): Atlantic Ocean. Approximate coordinates: 37°08' N and 32°22' W

- *Caulerpa taxifolia *(Chlorophyta) (origin Denpassar, Bali, Indonesia, purchased from Marinelife, Spijk, The Netherlands). Approximate coordinates: 8°41' S and 115°17' E.

Representative samples of about 5 g for each replicate were frozen in liquid nitrogen, ground to a fine powder using a laboratory mill (IKA, model A11B, Staufen, Germany) and stored at -80°C until analysis. Aliquots of about 250 mg of these powders were weighed and extracted with 4 ml chloroform/methanol (4:5, v/v), which contained glyceryl-triheptadecanoate, equivalent to 4 μg/ml of margarinic acid (C17:0), as an internal standard for quantification. After addition of 2.5 ml water and thorough mixing, phase separation was achieved by centrifugation. The methanol/water phase was washed twice with 1.0 ml chloroform. The combined chloroform phases, containing acyl lipids and other apolar compounds, were evaporated to dryness under a nitrogen stream. Hydrolysis and methylation of acyl lipids to fatty acid methylesters (FAMEs) were achieved by heating in 3 ml methanol, containing 5% concentrated sulphuric acid, for 3 h at 70°C. FAMEs were partitioned by phase separation into 3 ml *n*-hexane, which was washed twice with 1.0 ml water. Hexane fractions were passed over a small amount of water-free sodium sulphate to remove traces of water and acid before analysis on GCMS.

Aliquots (1 μl) were analysed on an Agilent 7890A gas chromatograph, connected to an Agilent 5975A mass selective detector, equipped with a ZB225-column (30 m length × 0.25 mm i.d. × 0.25 μm film thickness; Phenomenex, Utrecht, The Netherlands). The oven was programmed at an initial temperature of 60°C for 2 min, followed by heating at 20°C/min to 120°C, 3°C/min to 165, 2°C min to 215°C and a final time of 10 min. Helium was used as a carrier gas at 0.7 ml/min. The injector (splitless mode), interface, MS source and detector temperatures were 250°C, 290°C, 178°C and 290°C, respectively. Dry matter (DM)-based concentrations of fatty acids were calculated from fresh weight (FW)-based measurements through FW-to-DM conversion factors, determined after drying for 24 h at 60°C and then for one night at 105°C.

## Results

### Total acyl lipid concentration

Table 1 shows that *Ascophyllum nodosum *and *Fucus serratus *growing in the colder regions of the Atlantic, show the highest acyl lipid concentration with 45 and 37 mg per g dry matter (DM), respectively. *Ulva lactuca *and *Chondrus crispus*, which grow at the surface in the water column, have intermediate lipid concentrations of about 22 and 15 mg per g DM, respectively. Similar concentrations are observed for *Laminaria hyperborea, Undaria pinnatifida *and *Palmaria palmata *which are collected from the French and Irish region of the Atlantic Ocean. The tropical seaweeds *Caulerpa taxifolia *and *Sargassum natans *have the lowest concentrations of about 10 mg per g DM.

**Table 1:** Fatty acid (FA) composition of macroalgal species.

Fatty acid	n-Classification for unsaturated carbon	*Ulva lactuca*	*Chondrus crispus*	*Laminaria hyperborea*	*Fucus serratus*	*Undaria pinnatifida*	*Palmaria palmata*	*Caulerpa taxifolia*	*Ascophyllum nodosum*	*Sargassum natans*
		μg FA per g dry matter (% of total fatty acid)								
C14:0*		28 ± 13 (<1)	1170 ± 186 (8)	1297 ± 89 (7)	2354 ± 641 (6)	1163 ± 80 (6)	681 ± 56 (5)	236 ± 10 (2)	3027 ± 395 (7)	162 ± 24 (2)

C16:0*		2768 ± 808 (12)	2718 ± 186 (19)	3178 ± 173 (18)	7266 ± 1849 (19)	2935 ± 101 (16)	3500 ± 156 (25)	4942 ± 44 (39)	3693 ± 682 (8)	3006 ± 259 (41)

C16:1*	n-9	75 ± 61 (<1)	1512 ± 260 (10)	402 ± 57 (2)	566 ± 103 (1)	1147 ± 143 (6)	204 ± 47 (1)	443 ± 18 (4)	462 ± 74 (1)	325 ± 29 (4)

**C16:3**	n-3	119 ± 43 (1)	n.d.	n.d.	n.d.	n.d.	n.d.	1085 ± 45 (9)	n.d.	n.d.

**C16:4**	**n-3**	429 ± 8 (2)	n.d.	n.d.	n.d	n.d.	n.d.	n.d.	n.d.	n.d.

C18:0*		n.d.	n.d.	n.d.	316 ± 42 (1)	147 ± 6 (1)	133 ± 21 (1)	n.d.	240 ± 79 (<1)	369 ± 44 (5)

C18:1*	**n-9**	4502 ± 2204 (20)	2437 ± 296 (17)	3616 ± 53 (20)	15387 ± 6147 (41)	2339 ± 99 (13)	298 ± 59 (2)	882 ± 58 (7)	23193 ± 4833 (54)	1095 ± 147 (15)

**C18:1**	**unknown**	919 ± 707 (4)	212 ± 35 (1)	n.d.	76 ± 91(<1)	n.d.	179 ± 26 (1)	222 ± 17 (2)	120 ± 42 (<1)	74 ± 85 (1)

C18:2*	**n-6**	5548 ± 2108 (25)	312 ± 26 (2)	398 ± 37 (2)	3081 ± 906 (8)	800 ± 24 (4)	125 ± 40 (1)	n.d.	4884 ± 236 (11)	230 ± 29 (3)

C18:3*	**n-3**	4459 ± 865 (20)	233 ± 30 (2)	n.d.	1065 ± 149 (3)	1195 ± 65 (7)	312 ± 152 (2)	2161 ± 119 (18)	688 ± 19 (2)	121 ± 17 (2)

**C18:3**	**n-6**	484 ± 128 (2)	396 ± 21 (3)	n.d.	225 ± 68 (1)	243 ± 16 (1)	n.d.	n.d.	235 ± 42 (<1)	n.d.

**C18:4**	**n-3**	1846 ± 1648 (8)	1735 ± 282 (12)	2287 ± 92 (13)	547 ± 117 (1)	2255 ± 240 (12)	346 ± 111 (2)	354 ± 14 (3)	869 ± 354 (2)	n.d.

**C20:3**	**n-9**	n.d.	n.d.	n.d.	342 ± 116 (1)	n.d.	n.d.	n.d.	378 ± 46 (1)	n.d.

**C20:4**	**n-6**	355 ± 219 (2)	2158 ± 284 (15)	2095 ± 290 (12)	4775 ± 1150 (13)	2943 ± 155 (16)	332 ± 136 (2)	439 ± 38 (3)	4592 ± 2986 (10)	581 ± 81 (8)

C20:5*	**n-3, EPA**	234 ± 215 (1)	1216 ± 135 (8)	4781 ± 327 (26)	1424 ± 251 (4)	2858 ± 128 (16)	8339 ± 340 (59)	997 ± 87 (8)	1569 ± 127 (4)	329 ± 38 (5)

**C22:4**	**n-9**	n.d,	398 ± 116 (3)	n.d.	n.d.	n.d.	n.d.	n.d.	n.d.	n.d.

**C22:5**	**n-6**	610 ± 319 (3)	n.d.	n.d.	n.d.	n.d.	n.d.	n.d.	n.d.	n.d.

C22:6*	**n-3, DHA**	n.d.	n.d.	n.d.	n.d.	n.d.	n.d.	n.d.	n.d.	970 ± 109 (13)

**C24:0**		n.d.	n.d.	n.d	n.d.	n.d.	n.d.	156 ± 204 (1)	n.d.	n.d.

**Total**		**22387**	**14669**	**18054**	**37413**	**18191**	**14448**	**12592**	**44670**	**7262**

	**Total n-9**	21%	30%	22%	43%	19%	3%	11%	56%	19%

	**Total n-6**	32%	20%	14%	22%	21%	3%	4%	22%	11%

	**Total n-3**	32%	22%	39%	9%	35%	63%	38%	8%	20%

	**Ratio n-6: n-3**	**1.0**	**0.91**	**0.36**	**2.44**	**0.60**	**0.05**	**0.11**	**2.75**	**0.55**

Seven species were collected from the North Sea (*U. lactuca, C. crispus, L. hyperborea, F. serratus, U. pinnatifida, P. palmata, A. nodosum*) and two species from tropical seas (*C. taxifolia, S. natans*). Results are expressed as mean ± standard deviation in μg FA per g dry matter (n = 4) and, between brackets, as relative concentration (in % of total FA). FAs marked with * are identified on the basis of retention index similarity of authentic standards and of mass spectrum comparison with a Whiley library data base of their methyl esters. Unmarked FAs are identified on the basis of mass spectrum comparison with a Whiley library data base. Abbreviations: EPA = eicosapentaenoic acid (C20:5, n-3); DHA = docosahexaenoic acid(C22:6, n-3); n.d. = not detectable.

### Fatty acid composition

**Figure 2:**
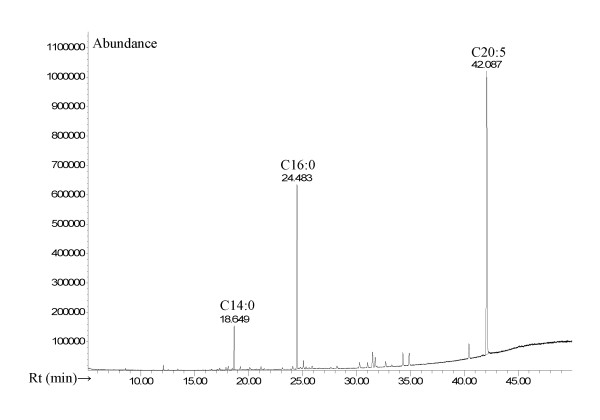
**GCMS pattern of fatty acid methyl esters of acyl lipids from the seaweed *Palmaria palmata***. Note the predominance of C14:0 (myristic acid, Rt = 18.65 min), C16:0 (palmitic acid, Rt = 24.48 min) and C20:5 (eicosapentaenoic acid = EPA, Rt = 42.09 min).

Palmitic acid (C16:0) was measured in all species at relatively high concentration. The lowest absolute value was measured in *C. crispus *(2.7 mg per g DM, 19% of total FA) and the highest absolute value in *F. serratus *(7.3 mg per g DM, 19% of total FA). The n-9 FAs were in the range of 3%-56% for *P. palmata *and *A. nodosum*, respectively. The important n-3 PUFA α-linolenic acid (C18:3) had the highest concentration in *U. lactuca *(4.5 mg per g DM, 20%) and another n-3 PUFA stearidonic acid (C18:4) had the highest absolute concentration in *L. hyperborea *and *U. pinnatifida *(2.3 mg per g DM, 12-13% of total fatty acids). The important "fish" n-3 PUFA eicosapentaenoic acid (EPA, C20:5) was the most abundant FA in *P. palmata *(8.3 mg per g DM, 59% of total FA, Figure 2).

A unique observation is the presence of another typical "fish" fatty acid, C22:6 at approximately 1 mg/g DM in *S. natans*.

The n-6 PUFA linoleic acid (C18:2) was found at the highest concentration of about 5 mg per g DM in *U. lactuca *and *A. nodosum*, while in *U. lactuca *also the highest proportion, 25% of total FA, was observed. Another n-6 PUFA, γ-linolenic acid, was only found in small amounts ranging from 1-3% of total FAs in some analyzed seaweeds. The n-6 FA arachidonic acid (C20:4) was found in the highest concentration in *F. serratus *and *A. nodosum *(about 4.5 mg per g DM, 13% and 10% of total FAs, respectively) and in *U. pinnatifida *(3 mg per g DM, 16%). The n-6: n-3 ratio in seaweeds was ≤ 1.0 for all species with the exception of *F. serratus *(2.44) and *A. nodosum *(2.75) (Table 1).

## Discussion

Lipid content has been described to be very low in seaweeds, ranging from 1 to 5% of dry matter and varies strongly between species [25], but their PUFA content can be as high as that of land plants [23,26] or even higher [27]. Fishery products and oil seeds contain much higher concentrations of PUFA over 20 and 50% of dry matter, respectively [2]. It should be noted that these PUFA supplies might become limiting in the near future. As stated in the introduction, the original source of long-chain PUFAs is not the fish itself, but marine algae and phytoplankton which form their major dietary source [20]. In the present study we also found lipid concentrations of 1-5% of dry matter for the nine seaweed species investigated and confirm that seaweeds can be a rich source of polyunsaturated fatty acids (PUFAs). They contain n-3 PUFAs such as C16:3 (isomer n-3), C16:4 (n-3), C18:3 (α-linolenic acid), C18:4 (stearidonic), and also long-chain n-3 PUFAs, e.g. C20:5 (EPA: eisosapentanoic acid) and C22:6 (DHA: docosahexaenoic acid). In addition, we found the following n-6 PUFAs: C18:2 (linoleic acid), C18:3 (γ-linolenic acid), C20:4 (arachidonic acid) and C22:5 (docosapentaenoic acid). These results corroborate with the results of other studies [27-30] although our study covered a larger variety of seaweed species.

One of the most interesting observations of this study was that the highest relative concentration of PUFAs in the red seaweed *Palmaria palmata *was observed for eicosapentaenoic acid (C20:5, EPA) accounting for 59% of the total fatty acid content. EPA is a very important n-3 FA in fish oil [19].

The total n-6 content was very low (only 3%) which corresponds to literature data [24,31] leading to a very low n-6: n-3 ratio (see Table 2). A low n-6: n-3 ratio is considered as a positive characteristic associated with prevention of inflammatory, cardiovascular and neural disorders. This may be a more general characteristic of red seaweeds because a similar result was observed in the edible red macroalga *Grateloupia turuturu *where the most abundant fatty acids were palmitic acid (C16:0) and EPA (C20:5) at proportions of 52% and 12%, respectively [32].

**Table 2:** Ratios of n-6: n-3 fatty acids in various seaweed species, as reported in earlier studies.

Seaweed species	Ratio n-6: n-3	Reference
*Porphyra *sp.	1.32	[35]

*Saccorhiza polyschides*	0.71	[24]
*Himanthalia elongata*	0.81-1.32	
*Laminaria ochroleuca*	0.83	
*Undaria pinnatifida*	0.49	
*Palmaria *sp.	0.13	
*Porphyra *sp.	1.21	

*Nereocystis luetkeana*	0.68	[36]
*Porphyra perforata*	0.46	
*Fucus distichus*	1.38	
*Fucus *sp.	1.49	
*Pterigophora *sp.	0.40	
*Ulva fenestra*	0.11	

*Porphyra *sp. from Japan, Korea	0.6	[37]
*Porphyra *sp. from China	1.8	
*Undaria pinnatifida*	0.5	
*Laminaria sp*.	1.3	
*Hizikia fusiforme*	0.3	

Hitherto, the margarine industry used both plants and seeds as the primary source of PUFAs. Seed oils, margarines and related products are rich in C18:2 and C18:3 [33]. n-6 PUFAs are mainly found in vegetable products. Linoleic acid (C18:2) is found in soybean, corn, nut, and sunflower oils, and γ-linolenic acid (C18:3) in borage, evening prim-rose and blackcurrant oils [2]. The major n-6 long chain PUFA is arachidonic acid (C20:4), which is found in egg yolk and muscle tissue [2]. The n-3 PUFA α-linolenic acid is also present in vegetable products (rapeseed, soybean and nut oils). The n-3 long chain PUFAs, e.g. C20:5 and C22:6, are mainly found in oily fish (e.g. mackerel, herring, and salmon) and fish oils [19], while smaller amounts are found in meat. DHA and its precursor EPA are common in marine fish and shellfish from cold waters [2,19]. Fish and shellfish from warmer marine or fresh water have ubiquitous DHA and EPA, however also the arachidonic acid (n-6) content is generally higher [2,19]. The n-6: n-3 ratio in the diet is currently recommended by the WHO to be less than10 [24] in order to prevent inflammatory, cardiovascular and neural disorders. The n-6: n-3 ratio found in our study for seaweeds is approximately 1.0. In addition, seaweeds are reported to also contain much lower concentrations of trans fatty acids than today's diet [3,34].

Table 2 (see also [24], [35]-[37]) gives an overview for the n-6: n-3 ratios of various seaweed sources, reported in the scientific literature. In our study we found several PUFAs in different concentrations in seven seaweed species from the North Sea (*Ulva lactuca, Chondrus crispus, Laminaria hyperborea, Fucus serratus, Undaria pinnatifida, Palmaria palmata, Ascophyllum nodosum*) and two tropical seaweeds (*Caulerpa taxifolia, Sargassum natans*). If we compare our data from Table 1 with other literature data of PUFAs in seaweeds, the following features arise:

- Green seaweeds like *Caulerpa *sp. and *Ulva *sp. are rich in C16:0 (palmitic acid) [38].

- Relatively high contents of polyunsaturated C16-fatty acids, which are uncommon in plants, were found in the green seaweeds *U. lactuca *and *C. taxifolia*. The C16 FAs were proposed to be taxonomically important among green macrophyte algae [39].

- These seaweeds also contained the essential fatty acids C18:2 (linoleic acid, n-6), C18:3 (α-linolenic acid, n-3), C20:4 (arachidonic acid, n-6) and C20:5 (eicosapentaenoic acid, n-3) [38]. For *Ulva *spp. from Turkey also high proportions of palmitic (C16:0), palmitoleic (C16:1), oleic (C18:1, n-9), linoleic acid (C18:2, n-6) and conjugated linolenic acids (C18:3, n-3 and n-6) were found [27]. In the study of El-Shoubaky et al. [27] the same compounds were found for *Ulva rigida *from Egypt and *U. fasciata *but in these species no C20:4 (n-6) and C20:5 (n-3) were found.

- Like reported in our study for *Sargassum natans*, palmitic (C16:0) and oleic acids (C18:1, n-9) were the most abundant FAs in *S. polycystum *[30]. However, in our study we found no docosapentaenoic acid (C22:5, n-6). Our observations for the PUFA content of *S. natans *correspond to those for other *Sargassum *spp. [30,40,41].

- For *Caulerpa taxifolia *we found low amounts of PUFAs. This is in agreement with Matanjun *et al*. [30] where in *C. lentillifera *also a low PUFA content in comparison to *Euchema cottonii *and *S. polycystum *was observed. In this study, *C. lentillifera *contained all the essential fatty acids linoleic acid (C18:2, n-6), α-linolenic acid (C18:3, n-3), and eicosapentaenoic acid (C20:5, n-3).

- In *Laminaria hyperborea *the highest concentrations were found for palmitic acid (C16:0), arachidonic acid (C20:4, n-6), oleic acid (C18:1, n-9), linoleic acid (C18:2, n-6) which is comparable to the observations of Dawczynski et al. [37]. In contrast to the study of Dawczynski et al. [37] we found no α-linolenic acid (C18:3, n-3).

- Characteristic for *Undaria pinnatifida *is the relatively high concentration of stearidonic acid (C18:4, n-3) which corresponds to earlier studies [37,42,43].

- In our study we investigated also two red seaweeds. Both had a totally different PUFA composition. *Chondrus crispus *has a high level of arachidonic acid (C20:4, n-6) which is in agreement with Fleurence *et al*. [43]. *Palmaria palmata *is a very interesting red seaweed. It contains eicosapentaenoic acid (EPA, C20:5, n-3) as the predominant fatty acid, and marginal concentrations of arachidonic acid (C20:4, n-6), linoleic acid C18:2, n-6). These data correspond to those found in earlier studies [37,42,43].

From the coherence between various studies on PUFA content between macroalgal species we can conclude that there is a strong genetic component in determining the PUFA content. The question remains to what extent the PUFA concentration and composition are determined by genus and environment [44,45].

Our study with seven seaweeds from the North Sea, each with a typical fatty acid composition, confirms that genetic factors are of major importance as also stated by Matanjun *et al*. [30] from a study with three tropical seaweeds and by Khotimchenko [29] with seven *Sargassum *species.

Other studies showed the effects of environmental factors, like temperature [36], annual cycle [32,46-48], salinity [49] and mineral content of the growth medium [50,51]. In *S. piluliferum *increasing salinity was associated with increasing levels of C18:4 (n-3), C20:4 (n-6), C20:5 (n-3), total n-3 and total n-6 PUFAs [49]. For *Ascophyllum nodosum*, the degree of fatty acid desaturation is associated with a lower water temperature, which is probably an adaptation of cell membranes to maintain their fluidity [27]. Also Colombo *et al*. [36] reported that the PUFA content and degree of desaturation of seaweeds harvested in cold regions (Canada) was higher than those harvested in tropical waters of southern China and the Indo-Pacific region [36]. In general, high contents of saturated fatty acids have been reported in warm water tropical seaweeds [40,52].

Our observations substantiate the conclusion that an appropriate choice of macroalgal species, given their high PUFA content and low n-6: n-3 ratio, may form a promising strategy to enhance food quality, e.g. to prevent inflammatory, cardiovascular diseases and nervous system disorders. Given the growing world population and increasing demand for qualitative and quantitative food supply, the present food sources, also for PUFAs, will almost certainly be insufficient in the near future [53]. Therefore, new sources have to be explored from which Integrated Multi-Trophic Aquaculture (abbreviated IMTA) is a strategy, which is presently being investigated for its economic and agronomic feasibility. Sea farming in marine off-shore systems of macroalgae forms part of this approach [54,55]. From these studies it becomes evident that general predictions on agricultural potential and economic cost-benefit balances are difficult to give, since they are strongly dependent on factors related to local circumstances, e.g. appropriate algal species, tidal range and scale size. Already at this stage of development a production for macroalgae of about 50 tons per ha has been achieved and this yield will increase when the potential of using off-shore farming seaweed agriculture is further optimized [56]. Because 70% of our globe is covered with ocean, seaweeds are a durable and virtually inexhaustible, additional source for PUFAs, but also of amino acids [37] and other useful bioactive ingredients [32,57], which are undeniably also accessible for developing countries.

## Conclusions

Marine macroalgae form a good, durable and virtually inexhaustible source for polyunsaturated fatty acids with an (n-6) FA: (n-3) FA ratio of about 1.0. This ratio is recommended by the World Health Organization to be less than 10 in order to prevent inflammatory, cardiovascular and nervous system disorders. Some marine macroalgal species, like *Palmaria palmata*, contain high proportions of the "fish fatty acid" eicosapentaenoic acids (EPA, C20:5, n-3), while in *Sargassum natans *also docosahexaenoic acid (DHA, C22:6, n-3) was detected. Especially EPA and DHA are crucial for proper development of the nervous system and prevention of cardiovascular diseases.

## List of abbreviations

DHA: docosahexaenoic acid; DM: dry matter; EPA: eicosapentaenoic acid; GCMS: Gas Chromatography Mass Spectrometry; PUFA: PolyUnsaturated Fatty Acid.

## Competing interests

The authors declare that they have no competing interests.

## Authors' contributions

All authors have read and approved the final manuscript. VvG is the leading scientist in this study. Together with HvK and WB he developed the concept of using marine macroalgae as source for long chain polyunsaturated fatty acids. VvG collected the samples of the macroalgal species investigated. VvG and JH contributed equally to the manuscript. JH coordinated the processing of plant material, the subsequent chemical analyses and interpretation of data. Together with VvG and WdV, JH performed the chemical analyses and developed the protocols for processing of plant material. JH and VvG contributed equally to the manuscript. WdV performed the majority of processing of plant material and contributed substantially in the protocol for taking representative samples of the heterogeneous plant material. He also performed GCMS analyses and primary data processing. HvK developed, together with VvG and WB, the concept of exploring the efficacy of using marine macroalgae for supply of long chain polyunsaturated fatty acids. WB is the principal investigator and responsible for research on application of marine sources for food and industrial purposes. Together with VvG and HvK he developed the concept of exploring the efficacy of using marine macroalgae for supply of long chain polyunsaturated fatty acids.
